# Uplift of the Tibetan Plateau driven by mantle delamination from the overriding plate

**DOI:** 10.1038/s41561-024-01473-7

**Published:** 2024-07-02

**Authors:** Yuan Xie, Attila Balázs, Taras Gerya, Xiong Xiong

**Affiliations:** 1 School of Geophysics and Geomatics, China University of Geosciences, Wuhan, China; 2 Department of Earth Sciences, Institute of Geophysics, ETH Zurich, Zurich, Switzerland

**Keywords:** Geodynamics, Tectonics

## Abstract

The geodynamic evolution of the Tibetan Plateau remains highly debated. Any model of its evolution must explain the plateau’s growth as constrained by palaeo-altitude studies, the spatio-temporal distribution of magmatic activity, and the lithospheric mantle removal inferred from seismic velocity anomalies in the underlying mantle. Several conflicting models have been proposed, but none of these explains the first-order topographic, magmatic and seismic features self-consistently. Here we propose and test numerically an evolutionary model of the plateau that involves gradual peeling of the lithospheric mantle from the overriding plate and consequent mantle and crustal melting and uplift. We show that this model successfully reproduces the successive surface uplift of the plateau to more than 4 km above sea level and is consistent with the observed migration of magmatism and geometry of the lithosphere–asthenosphere boundary resulting from subduction of the Indian plate and delamination of the mantle lithosphere of the Eurasian plate. These comparisons indicate that mantle delamination from the overriding plate is the driving force behind the uplift of the Tibetan Plateau and, potentially, orogenic plateaus more generally.

## Abstract (web summary)

Delamination of the lithospheric mantle from the overriding Eurasian plate below the Tibetan Plateau is consistent with topographic, magmatic and seismic observations, according to numerical simulations of the geodynamic evolution of the plateau.

## Main

**Fig. 1:**
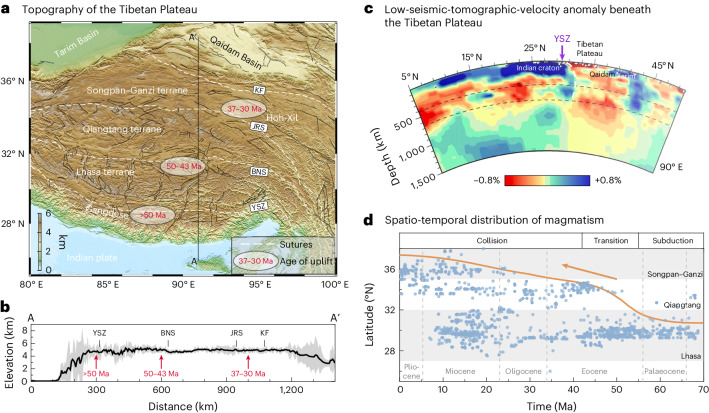
Topography, seismic structure and magmatic evolution of the Tibetan Plateau. **a**, Topographic map of the Tibetan Plateau. The red numbers indicate the ages of uplift at different locations (same in **b**)^6,41,45,46^. YSZ, Yalung–Zangpo suture; BNS, Bangong–Nujiang suture; JRS, Jinsha River suture; KF, Kunlun fault. Suture zone data from ref. ^47^. **b**, The swath profile shows the extreme elevations in a 100-km-wide area along profile A–A′. **c**, Seismic tomographic cross-section perpendicular to the collision front at 90° E modified from ref. ^17^. The dashed white line shows the inferred overturned Indian slab and the south-dipping lithospheric mantle of the Qaidam Basin. **d**, The spatio-temporal distribution of magmatic rocks across the plateau. Magmatism data are from ref. ^48^. The orange line and arrow show the inferred migration of magmatism.

The Tibetan Plateau is a geological marvel resulting from the collision of the Indian and Eurasian continents (Fig. 1a). Spanning over 2.5 million km^2^, this plateau reaches an average elevation exceeding 4 km, cradling within it the world’s thickest crust, which extends down to a depth of 80 km (refs. ^1–3^). This unique landscape is shaped by the interplay between tectonic plate interactions, crustal deformation and mountain-building processes, which requires the exploration of geodynamic processes and related surface fingerprints, including topographic evolution and magmatic distribution throughout its growth history. However, the mechanisms controlling the formation and preservation of such extreme topographic features remain widely debated.

Various reconstructions have been proposed regarding the topographic evolution of the plateau. Previous models suggested the northwards stepwise growth of Tibet from the Eocene driven by the indentation of India deep into Asia^4–6^. Other views hypothesized the presence of a high proto-plateau in central Tibet, inherited from the preceding subduction and collision history that expanded southwards and northwards mainly from the Miocene onwards^7,8^. Another model suggests that central Tibet was once a low-topographic valley lying between two high mountainous areas to the south and north. This central valley is thought to have attained its high elevation in the Late Oligocene driven by mantle upwelling and crustal shortening^9,10^. These palaeo-altimetry studies indicate that the Tibetan Plateau has grown in several successive phases (Fig. 1a,b). However, the reasons behind this progressive uplift pattern across the entire plateau remain uncertain.

Seismic tomography studies have identified a low-velocity anomaly beneath central Tibet, commonly attributed to an elevated, hot and partially molten asthenosphere^1,11,12^ or, alternatively, a metasomatized lithospheric mantle^13^. Gravity data inversion further supports these findings from seismic investigations, suggesting a shallow asthenospheric upwelling beneath central and northern Tibet, and partially beneath southern Tibet^14,15^. Recent tomography results show that the low-seismic-velocity zone varies along the strike, expanding from south to north^16,17^. Additionally, tomographic images reveal the overturned geometry of the cold Indian slab and a south-dipping positive velocity anomaly beneath the northern Tibet^17^ (Fig. 1c).

**Extended Data Fig. 1:**
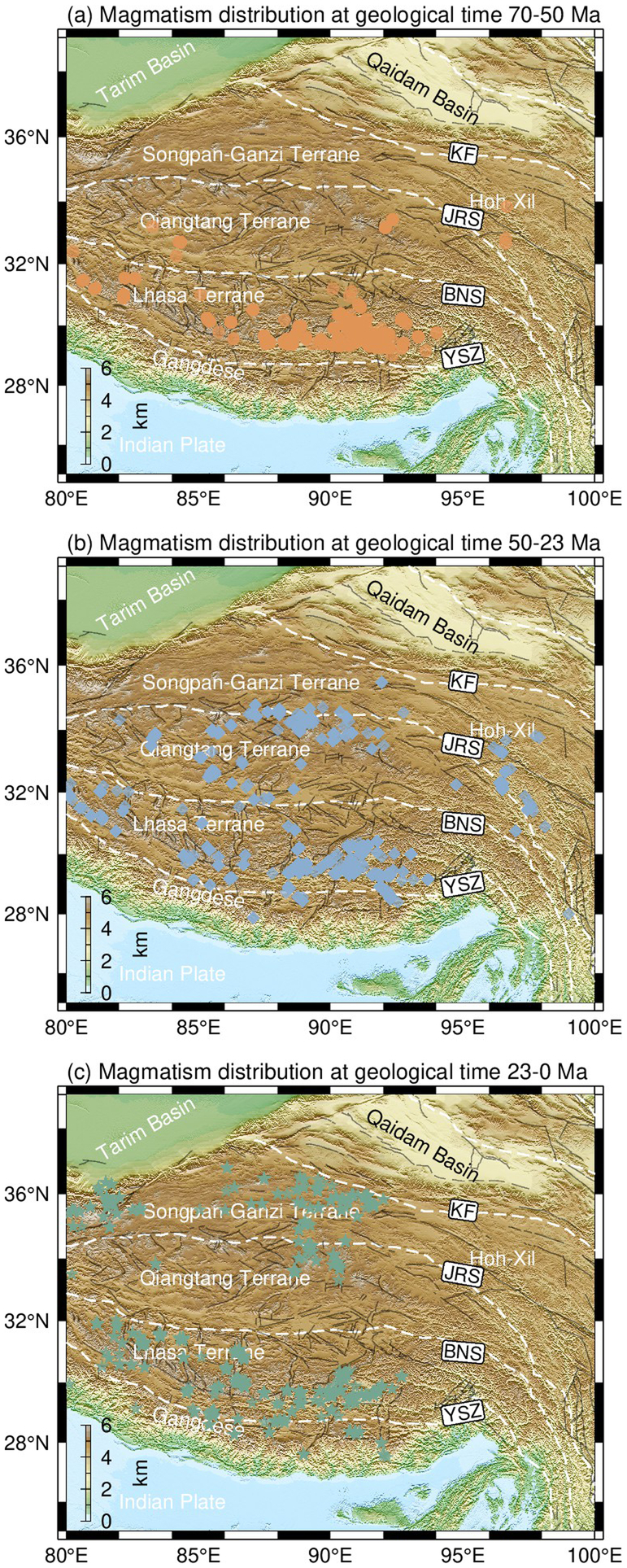
Distribution of magmatism during different periods on the Tibetan Plateau. **a**, Magmatism distribution at 70–50 Ma mainly constrained in Lhasa terrane. **b**, Magmatism distribution from 50 to 23 Ma migrated to northern Qiangtang terrane. **c**, Magmatism distribution at 23–0 Ma reaching north of Songpan-Ganzi terrane. Magmatism data is from Ref.^48^.

The spatio-temporal distribution of magmatism provides an important constraint for understanding the crustal and mantle processes of the Tibetan Plateau (Fig. 1d and Extended Data Fig. 1). Magmatism predating 50 Ma was associated with the subduction arc^18^. A transition in geochemical composition between 55 and 45 Ma is interpreted as a result of the transition from subduction to collision^18–21^. From the Eocene onwards, magmatic activity has been documented in both the Lhasa and Qiangtang terranes^18,22–24^. Subsequently, from the Miocene, magmatism migrated northwards to the Songpan–Ganzi terrane^22,25^. Several mechanisms have been proposed to explain the origin of post-collisional magmatism on the plateau, including intra-plate continental subduction of the Songpan–Ganzi lithosphere beneath the Qiangtang terrane^22,24,26^, convective thinning and delamination of the lithospheric mantle^19,22,27^, continental subduction^28^ and slab break-off events^20^. Although the pattern and origin of magmatism remain debated, at a first order, the post-collisional magmatic rocks in the plateau appear to be younger in the north (Fig. 1d and Extended Data Fig. 1).

Various models and geodynamic processes have been proposed to explain different aspects of plateau growth. Seismological studies inferred the shallow underthrusting of the strong Indian crust and lithospheric mantle beneath the Himalayas and southern Tibet^3^. However, this model of underthrusting thick and cold lithosphere below Tibet cannot explain the observed low-seismic-velocity anomaly beneath the plateau^11,12,16,17^, and the south-dipping Asian slab in the north^29^. Furthermore, the migration of magmatism requires additional driving factors. Tomographic data^30^ and Eocene magmatism in the Qiangtang terrane^22,24^ have been attributed to intra-plate continental subduction of the Asian lithosphere beneath the Jinsha River suture^4^. This transmission of compressional stresses far from the collision front may explain plateau widening^31^. Alternatively, the Eocene inward-dipping intracontinental subduction of both the Lhasa terrane and Eurasian lithosphere beneath central Tibet is hypothesized on the basis of palaeo-altimetry studies, followed by the detachment of the Lhasa lithospheric mantle^10^. Previous models have also demonstrated that convective removal of the lithospheric mantle following crustal shortening, resulted in 1–3 km surface uplift of the plateau^27,32^. Another plausible explanation is delamination process^33–35^. However, classical post-collisional mantle delamination models involving the decoupling and sinking of lithospheric mantle from the overlying crust typically assume the existence of a weak zone often linked to a preceding subduction forearc region connected to the upwelling of low-density melts and eventually control the topographic uplift of the lower plate^33,36–38^. Geological and geophysical findings have established a connection between successional growth, magmatic activity and lithospheric mantle removal processes. Nevertheless, a unified, self-consistent model that incorporates all these first-order observations is missing thus far.

In this Article, we present a thermo-mechanical numerical model of the Tibetan-style orogeny that provides an integrated explanation for diverse observations on the tectono-magmatic evolution of the plateau. Our model introduces the concept of overriding plate mantle delamination with the gradual removal of lithospheric mantle from the overriding plate induced by variable subduction velocities and related melting. The delamination process governing orogenic plateau formation provides a genetic link between magmatic migration and the migration of delamination fronts under thickened continental crust. This model challenges the conventional perspective on the formation of the Tibetan Plateau and opens avenues for understanding the formation of orogenic plateaus and related migration of magmatism.

## Evolution of the delamination process

**Fig. 2:**
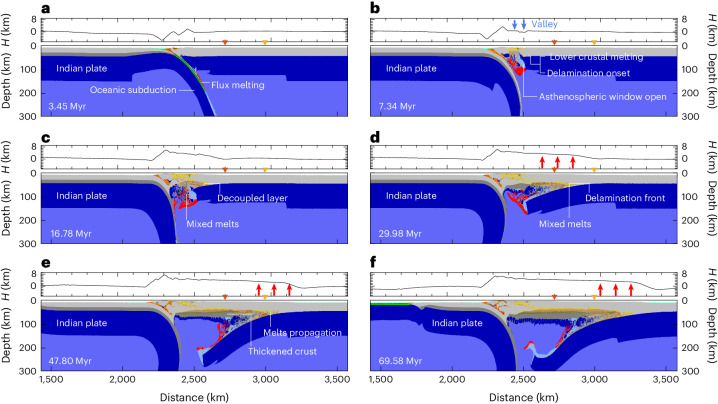
Evolution of overriding plate mantle delamination shown by the modelled topography and rock composition. **a**, Transition from subduction to collision. **b**, Opening of the asthenospheric window and onset of delamination. **c**, Intrusion of melts into the Moho, and peeling-off of the lithospheric mantle from the overlying crust. **d**, A mixture of mantle and crustal melts moves laterally along the Moho. The red arrows show the corresponding surface uplift. **e**, Ongoing delamination widens the orogeny. Crustal thickening is due to Indian continental subduction. **f**, The mature stage of mantle delamination. The overall collision system features two downgoing lithospheric mantle structures, including continental subduction of the Indian slab and delamination of the Eurasian lithospheric mantle. The topographic evolution of locations marked by orange and yellow triangles is shown in Fig. 4c,d. *H*, modelled topographic height.

Subduction of the Indian slab initiates a series of linked tectonic and magmatic processes. Subduction of the oceanic slab hydrates the overlying mantle wedge and controls flux melting (Fig. 2a). The resulting mixture of hydrated, serpentinized and partially molten mantle gradually ascends towards the overlying crust, leading to thermo-rheological weakening (Fig. 2b). The gradual rise and emplacement of melt products lead to thinning of the lithosphere, which is connected to increasing mantle and crustal temperatures (Fig. 2b,c). This process enhances further accumulation of melts in the arc and back-arc regions, creating an asthenospheric window where melts are focused. In the volcanic arc and its adjacent back-arc region, the crust undergoes thinning, leading to the formation of a valley around 75 km wide (Fig. 2b).

As melts amass and laterally intrude along the Moho interface, the lithospheric mantle decouples from the overlying lower crust, leading to the onset of overriding plate mantle delamination (Fig. 2b,c). The mixture of mantle flux melting and crustal melting can be compared with the Palaeocene to Middle Eocene magmatic activity in southern Tibet (Fig. 1d). Subsequently, the orogenic region gradually expands, giving rise to a proto-plateau with a high elevation (>4 km) spanning ca. 500 km (Fig. 2c).

Overriding plate mantle delamination is facilitated and maintained by the lateral migration of partially molten mantle and crustal rocks beneath and above the Moho. As the mantle lithosphere gradually peels off, the overlying surface records uplift, leading to the widening of the proto-plateau (Figs. 2d–f and 4a). Before delamination, the proto-plateau was affected by ca. 1–3 km of uplift driven by crustal shortening. As the lithospheric mantle is replaced by hot, partially molten and low-density asthenosphere, the orogenic region is uplifted by an additional ca. 2 km. Eventually, a more than 1,000-km-wide orogenic plateau formed, maintaining an average elevation of ca. 4 km (Figs. 2f and 4a,b). During the ongoing continental subduction stage, the upper crust of the downgoing Indian plate is exhumed to the surface, contributing to the rise of the frontal orogen, whereas the lower crust is partly relaminated beneath the orogen. These processes result in thickening of the crust within the orogenic core.

**Fig. 3:**
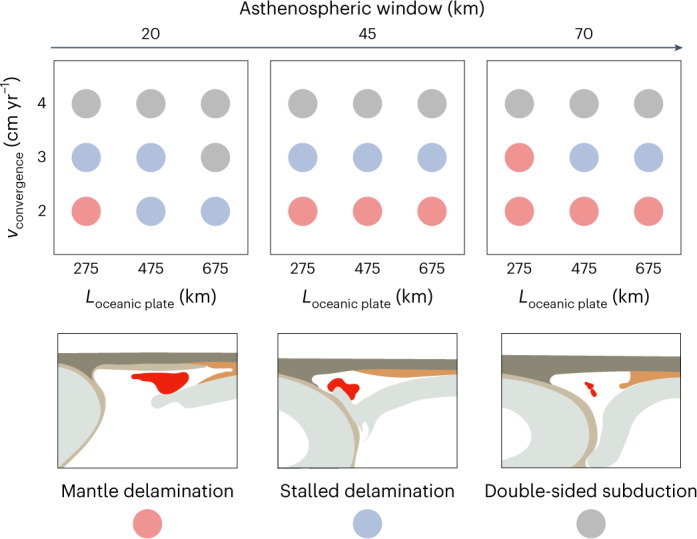
Regime diagram of the main controlling parameters. The three controlling parameters include the convergence rate (*v*_convergence_), the length of the oceanic plate (*L*_oceanic plate_) and the thickness of the asthenospheric window. The models are grouped into distinct scenarios according to their evolution by 40 Myr.

**Extended Data Fig. 2:**
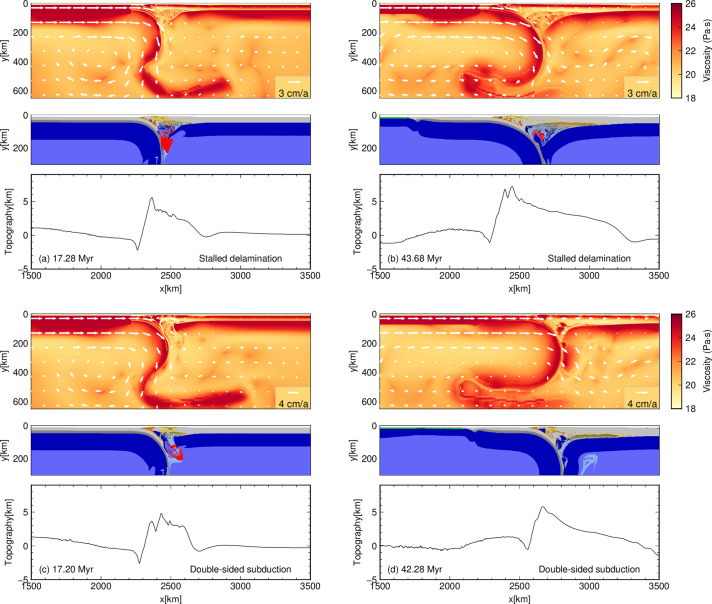
Model predictions of alternative lithospheric mantle delamination experiments. (a) and (b): Stalled delamination; (c) and (d): Double-sided subduction^39^.

Depending on the explored variations in the length of the oceanic plate, thickness of the asthenospheric window and convergence rate, the investigated model series can be grouped into three end-member scenarios based on their lithospheric evolution: (1) mature delamination, (2) stalled delamination and (3) double-sided subduction^39^ (Fig. 3). In the latter two scenarios, the advancing Eurasian slab prevents the overriding plate mantle from sinking, or the lithospheres become coupled at depth (Extended Data Fig. 2). The competition between the advance of the subducting plate and the sinking of the overriding plate lithosphere governs whether a prolonged delamination and melting stage was reached (Fig. 2). However, in the latter two scenarios, this system lacks sufficient time and space to trigger substantial asthenospheric upwelling, thereby preventing lateral propagation of the delamination front and related mantle and crustal melting.

## Comparison between models and observations

**Fig. 4:**
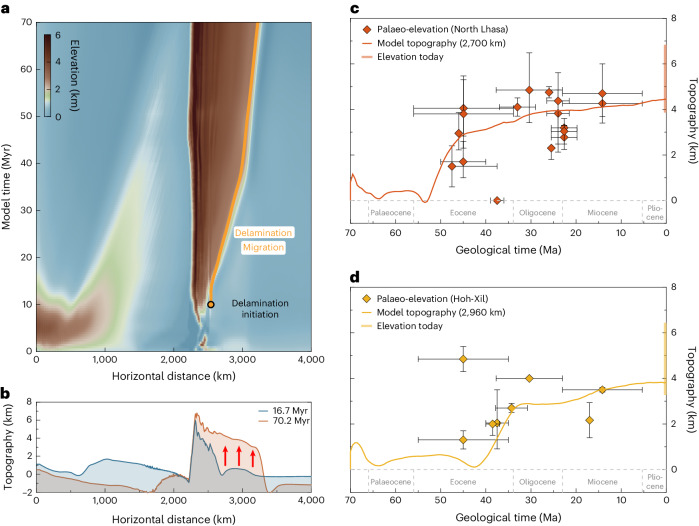
Topographic evolution of the overriding plate mantle delamination model and comparisons with palaeo-altitude studies. **a**, Topographic temporal evolution along the 2D model profile. The orange line indicates the spatial propagation of delamination. The black circle indicates the onset of delamination. **b**, Topography profiles at different numerical times. The red arrows show the uplift of the topography and the transition from a proto-plateau to a wide, elevated plateau. **c**,**d**, The diamonds with error bars show the palaeo-elevations in the northern Lhasa and Hoh-Xil Basin (palaeo-elevations are available in Supplementary Table 3)^6,41,45,46^. The solid lines indicate the topographic evolution of different locations, as shown in Fig. 2, corresponding to the northern Lhasa and Hoh-Xil Basin, respectively. **c**, Comparison between modelled topographic variation and paleo-elevations in northern Lhasa terrane. **d**, Comparison between modelled topographic evolution and palaeo-elevations in Hoh-Xil Basin. Source data

The overriding plate mantle delamination model offers a comprehensive explanation for the progressive uplift and gradual widening of the orogenic plateau, driven by the propagation of melts along the Moho (Fig. 2). Palaeo-altitude studies of the Tibetan Plateau have indicated gradual growth over millions of years in response to the collision between the Indian and Eurasian continental plates^4,6,40^. Our modelled surface evolution can be compared with reconstructions (Fig. 4). Figure 4c shows the modelled temporal topographic variation at the location *x* = 2,700 km, alongside the corresponding palaeo-elevations of central Tibet. The model shows that central Tibet experienced rapid uplift within 8 Myr, reaching 3 km in elevation (geological ages 53–45 Ma), followed by a gradual increase to ca. 4 km. These results are substantiated by the palaeo-elevation studies, which support that central Tibet experienced a rise in the Eocene^6,10,41^, and reached 4 km by the Oligocene (Fig. 4c)^6,10^.

In Fig. 4d, the modelled topographic evolution at the location *x* = 2,960 km corresponds to the Hoh-Xil Basin in central to northern Tibet. The model suggests that the Hoh-Xil Basin was at a low relief in the Middle Eocene, which aligns well with the palaeo-elevation data indicating an elevation of less than 2 km during that period^42^. Subsequently, the model results suggest that the Hoh-Xil Basin uplift began in the Late Eocene, with gradual uplift reaching an elevation of ca. 4 km by the Miocene. The dating results indicate that the Hoh-Xil Basin experienced gradual uplift during the Late Eocene and Early Oligocene and eventually reached an elevation of ca. 3.5 km by the Miocene^41^. While the uncertainty of the uplift history may be linked to neglecting three-dimensional (3D) effects, the role of elasticity and the use of simplified surface processes, the model results demonstrate good agreement with the palaeo-altitude estimates in terms of spatial patterns and uplift values. In addition, delamination was initiated beneath the weak volcanic arc formed during oceanic subduction. Inherited rheological weak suture zones in the overriding plate, which may alter the location and distribution of intra-plate deformation^10,31^, are not defined in our models.

**Fig. 5:**
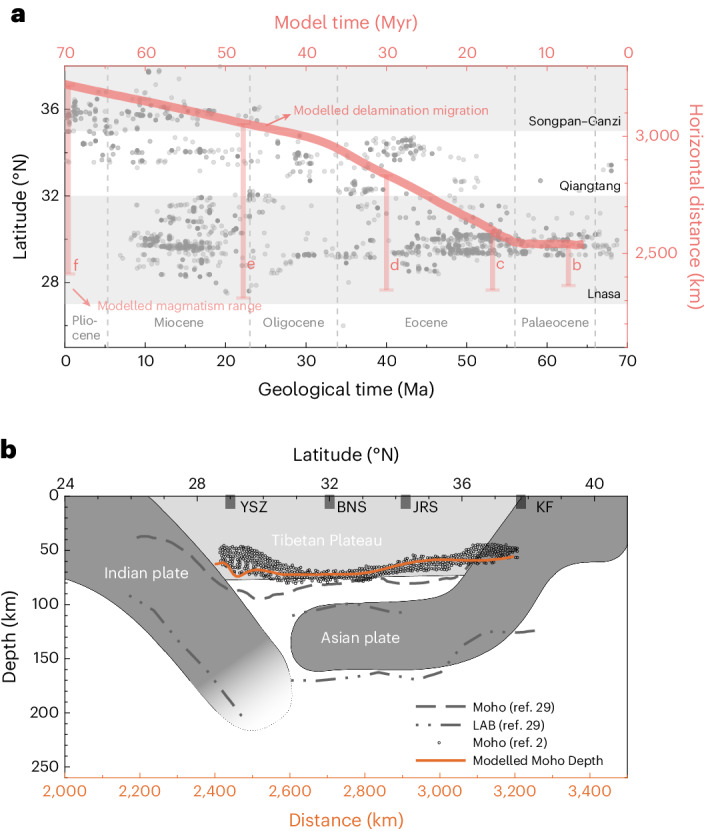
Comparison of the model results with magmatism and seismological data. **a**, The distribution of magmatic rocks on the Tibetan Plateau, marked by grey dots. Magmatism data from ref. ^48^. The red line shows the modelled delamination migration linked to the movement of melts. The vertical light-red lines show the modelled magmatism range (that is, the spatial occurrence of molten rocks in the reference model) at different evolution stages in Fig. 2. **b**, The deep structure beneath the Tibetan Plateau revealed by S-wave receiver functions. The cartoon is modified from ref. ^29^. YSZ, Yalung–Zangpo suture; BNS, Bangong–Nujiang suture; JRS, Jinsha River suture; KF, Kunlun fault. Source data

The presented mantle delamination model can be compared with the spatio-temporal distribution of post-collisional magmatism in Tibet. Our numerical models highlight that the propagation of the lithospheric delamination front under the growing Tibetan Plateau is genetically associated with the migration of magmatism (Figs. 2 and 5a). This is well supported by the observed first-order northward migration of magmatism from the collision front towards the continental hinterland (Figs. 2c–f and 5a). The simulated magmatism in the Palaeocene and Eocene (Fig. 2b,c) can be compared with the earliest magmatism during the transition from oceanic subduction to continental collision in the Lhasa terrane^18–21^. Subsequently, the modelled magmatism migrates further north from the Late Eocene to the Miocene (Fig. 2d,e). The Early Eocene volcanism of the Qiangtang block was connected to the reactivation of inherited sutures bounding the Lhasa and Songpan–Ganzi terranes leading to intra-plate continental subduction and related mantle upwelling and melting^4,22,24^. The role of such inherited suture zones can be tested in future studies. The most recent volcanism that formed during the Palaeocene and Miocene in the Songpan–Ganzi terrane^43^ is also consistent with the modelled magmatic pattern (Figs. 2f and 5a).

Furthermore, the removal of strong and cold lithosphere and its replacement with hot asthenospheric mantle, as simulated in the model, are consistent with available seismic tomographic models that show slow seismic velocities beneath the Tibetan Plateau^11,12,16,17^. The modelled crustal thickness is compared with the estimates from receiver function studies (Fig. 5b). Beneath southern and central Tibet, the crust is extremely thickened, reaching ca. 70–80 km in depth, while the the crustal thickness of the northern part is ca. 40–60 km (ref. ^2^). The estimated crustal thickness inferred from our numerical model ranges between 55 km and 74 km, with the thickest crust occurring at the centre of the modelled plateau geometry and gradually decreasing northwards. These results are consistent with the estimates from receiver functions^2^, suggesting that parts of the thickened crust may have originated from underplating continental crustal material from the Indian slab. In particular, the deep bivergent structure of the Tibetan Plateau with southward-dipping Eurasian mantle in the north^17,29,44^ and northward subduction of the Indian continental plate revealed by receiver function images and tomographic models^16,29^ reconciles the predicted lithospheric structure in the delamination model (Fig. 5b).

To conclude, the presented numerical modelling results provide compelling evidence that overriding plate mantle delamination associated with the upwelling and melting of hot asthenosphere is the key driver of the gradual widening and growth of orogenic plateaus. This process has shaped the Indian–Eurasian plate collision, coupled with voluminous magmatism throughout the uplift history of the plateau. The delamination model successfully captured the key features of the Tibetan Plateau, including its lithospheric structure with two downgoing mantle anomalies, as revealed by geophysical data and the migration of magmatism. The comparison suggests that overriding plate mantle delamination may be a common yet ‘hidden’ geodynamic process associated with major magma-rich orogenic episodes throughout the Earth’s history.

## Methods

We used the two-dimensional (2D) thermo-mechanical code I2VIS^49^. This code solves the momentum, continuity and heat conservation equations based on marker-in-cell and finite-differences methods:

1 $$\frac{\partial {\sigma }_{ij}^{{\prime} }}{\partial {x}_{j}}-\frac{\partial P}{\partial {x}_{j}}+\rho {g}_{i}=0$$

2 $${\mathrm{div}}(\overrightarrow{\bf v})=\frac{\partial {v}_{x}}{\partial x}+\frac{\partial {v}_{y}}{\partial y}=0$$

3 $$\rho {C}_{P}\left(\frac{{\mathrm{D}}T}{{\mathrm{D}}t}\right)=\frac{\partial }{\partial {x}_{j}}\left(\kappa \frac{\partial T}{\partial {x}_{j}}\right)+{H}_{{\mathrm{a}}}+{H}_{{\mathrm{r}}}+{H}_{{\mathrm{s}}},$$

where ${\sigma }_{ij}^{{\prime} }$ is the deviatoric stress tensor, *P* is the pressure, *ρ* is the density, *g*_*i*_ is the gravitational acceleration, *v*_*x*_ and *v*_*y*_ are the horizontal and vertical velocity components, respectively, *C*_*P*_ is the isobaric heat capacity, D*T* and D*t* are the substantive time derivatives, *T* is the temperature, *κ* is the thermal conductivity coefficient and *H*_a_, *H*_r_ and *H*_s_ are the adiabatic, radioactive and shear heat production, respectively.

### Rheology

The rheology used in this study is visco-plastic. The viscous creep of rocks is defined in terms of deformation invariants and depends on temperature, pressure and strain rate. We consider the plastic and viscous rheology of rocks, which behave like slowly creeping fluids at a long time scale. The plastic rheology follows the Drucker–Prager yield criterion^50^:

4 $${\sigma }_{{{{\rm{yield}}}}}=C+P{\mathrm{sin}}(\varphi )$$

5 $$P{\mathrm{sin}}(\varphi )=P{\mathrm{sin}}({\varphi }_{{{{\rm{dry}}}}})(1-\lambda )$$

6 $${\eta }_{{{{\rm{plastic}}}}}=\frac{{\sigma }_{{{{\rm{yield}}}}}}{2{\dot{\varepsilon }}_{{\mathrm{II}}}},$$

where *σ*_yield_ is the yield stress, *C* is the cohesion, *φ* is the internal friction angle (*φ*_dry_ stands for dry rocks), *λ* is the pore fluid pressure factor and ${\dot{\varepsilon }}_{{\mathrm{II}}}$ is the second invariant of the strain rate. The viscous rheology takes the form^50^

7 $${\eta }_{{{{\rm{ductile}}}}}={A}_{{\mathrm{D}}}^{-1/n}{\sigma }^{\,(1-n)/n}{{\mathrm{e}}}^{\frac{{E}_{{\mathrm{a}}}+P{V}_{{\mathrm{a}}}}{nRT}},$$

where *E*_a_ is the activation energy, *V*_a_ is the activation volume, *n* is the stress exponent, *R* is the gas constant and *A*_D_ is a material constant. Finally, the minimum of plastic and ductile viscosities defines the effective viscosity

8 $${\eta }_{{{{\rm{eff}}}}}={\mathrm{min}}({\eta }_{{{{\rm{plastic}}}}},{\eta }_{{{{\rm{ductile}}}}}).$$

### Melts

The melt fraction is assumed by a linear function of temperature in the calculation. For a given pressure and rock type, the volumetric melt fraction *M* is calculated using the relationship^51^

9 $$\left\{\begin{array}{l}M=0,{{\mbox{}}}T\le {T}_{{{{\rm{solidus}}}}}\quad \\ M=\frac{T-{T}_{{{{\rm{solidus}}}}}}{{T}_{{{{\rm{liquidus}}}}}-{T}_{{{{\rm{solidus}}}}}},{{\mbox{}}}{T}_{{{{\rm{solidus}}}}} < T < {T}_{{{{\rm{liquidus}}}}}\quad \\ M=1,{{\mbox{}}}T\ge {T}_{{{{\rm{solidus}}}}}\quad \end{array}\right.,$$

where *T*_solidus_ and *T*_liquidus_ are the solidus and liquidus temperatures, respectively, of the given lithology (Supplementary Table 1).

The effective density (*ρ*_eff_) of partially molten rocks changes with the melt fraction and *P*–*T* conditions:

10 $${\rho }_{{{{\rm{eff}}}}}={\rho }_{{{{\rm{solid}}}}}-M(\,{\rho }_{{{{\rm{solid}}}}}-{\rho }_{{{{\rm{molten}}}}})$$

11 $${\rho }_{P,T}={\rho }_{0}[1-\alpha (T-{T}_{0})][1+\beta (P-{P}_{0})],$$

where *ρ*_solid_ and *ρ*_molten_ are the densities of the solid and molten rock, respectively, *ρ*_0_ is the standard density at *P*_0_ = 0.1 MPa and *T*_0_ = 298 K, and *α* and *β* are the thermal expansion coefficient and compressibility coefficient, respectively.

After partial melting occurs in rocks (*M* > 0), the effective heat capacity (*C*_pe_) and thermal expansion coefficient (*α*_e_) also increase accordingly:

12 $${C}_{{\mathrm{pe}}}={C}_{P}+{Q}_{L}[{(\partial M/\partial T\;)}_{P = {{{\rm{const}}}}}]$$

13 $${\alpha }_{{\mathrm{e}}}=\alpha +\rho {Q}_{L}[{(\partial M/\partial P)}_{T = {{{\rm{const}}}}}]/T$$

### Model setup

The model simulates the forced subduction of an oceanic plate and subsequent continental collision, and is based on an area of 4,000 km × 2,000 km (Supplementary Fig. 1). The rectangular grid with 1,361 × 351 nodes is non-uniform, including a higher resolution (1 km × 1 km) in the 1,000-km-wide region in the centre, while the rest of the models are resolved by coarser resolution (from 1 km × 1 km to 10 km × 10 km). The models contain a continental upper plate, while the lower plate includes an oceanic domain and a continental domain. The widths of the oceanic plate are 275 km, 475 km and 675 km in different model series bounded by wide, thinned passive margins. The oceanic crust is composed of 3 km of basalts and 5 km of gabbros. The overriding continental plate consists of 35 km of felsic crust^52^, while the continental lower plate includes 20 km of felsic crust and 15 km of mafic crust. The mantle is composed of dry olivine. The detailed material physical properties can be found in Supplementary Table 1. The setup includes a hot and weak forearc region connected to the long-lasting subduction history preceding collision^53–55^. Consequently, a weak and hot mantle wedge and forearc regions are considered and implemented as an asthenospheric window in the setup^56,57^.

A temporally variable internal convergence velocity is applied on the subducting plate to drive the whole subduction–collision system, while no velocity is applied on the overriding plate. The initial velocity is 10 cm yr^−1^, followed by a decrease to 4.5 cm yr^−1^ at the beginning of continental collision, and a gradual decrease to 4 cm yr^−1^, 3 cm yr^−1^ or 2 cm yr^−1^ in different model series. Plate convergence velocities during subduction and collision were adapted from plate reconstruction studies^58,59^. All kinematic boundary conditions are free slip, except for the lower boundary, which is permeable and implies an infinity-like external free slip condition^51^. To simulate the topographical evolution, a 10-km-thick ‘sticky air’ layer with a viscosity of 10^18^ Pa s and a density of 1 kg m^−^^3^ is placed on top of the model as the internal free surface.

A stronger lower plate representing the cold Indian cratonic lithosphere and a weaker upper plate with hot, felsic crust indicating the weak Eurasian plate were used in the model^52^. A strong zone with a width of 900 km on the right side of the model domain was set to represent the stable Tarim–North China lithosphere. Different thermal gradients have been used for different model domains. For the young overriding plate, the temperature is set to 0 °C at the surface, rises to 530 °C at the Moho depth, and further increases to 1,350 °C at the base of the continental lithosphere at a 140 km depth. For the strong Tarim–North China lithosphere, the temperature increases linearly from the surface (0 °C) to 1,027 °C at a 140 km depth. For the Indian plate, the temperature increases from the surface (0 °C), to 600 °C at a depth of 20 km, reaches 670 °C at the Moho, and then rises to 1,027 °C at a depth of 140 km. An adiabatic thermal gradient of 0.5 °C km^−1^ is used for the asthenosphere.

### Model limitations

Our 2D models neglect any along-strike variations, including complex 3D mantle flow effects, oblique plate geometries and the along-strike variations of collision or break-off. Nevertheless, the presented 2D models show good agreement with the first-order observations, including successive stages of the plateau uplift, the northward trend of magmatism, the low velocity anomaly beneath the plateau, the northward-subducting Indian plate and the southward-dipping Asian slab. However, the detailed evolution of the magmatism distribution and the exact uplift events are not fully solved.

## Online content

Any methods, additional references, Nature Portfolio reporting summaries, source data, extended data, supplementary information, acknowledgements, peer review information; details of author contributions and competing interests; and statements of data and code availability are available at 10.1038/s41561-024-01473-7.

### Supplementary information

Supplementary Information Supplementary Figs. 1–6, Tables 1–3 and references.

### Source data

Source Data Figs. 4 and 5 Modelling results for Figs. 4 and 5.

## Data Availability

All the relevant data and model outputs presented in this study are available via Zenodo at 10.5281/zenodo.10426375 (ref. ^60^). Source data are provided with this paper.

## Code availability

The numerical code used to conduct the geodynamic simulations is available via Zenodo at 10.5281/zenodo.10426375 (ref. ^60^).
