# Supplementary material for: Uplift of the Tibetan Plateau driven by mantle delamination from the overriding plate
Source: Nat Geosci. 2024 Jul 2;17(7):683–8. doi: 10.1038/s41561-024-01473-7 (PMC11245390; doi:10.1038/s41561-024-01473-7)
Supplement: Supplementary file 1 — Supplementary Figs. 1–6, Tables 1–3 and references. [file 41561_2024_1473_MOESM1_ESM.pdf]

nature geoscience

Supplementary information https://doi.org/10.1038/s41561-024-01473-7

Uplift of the Tibetan Plateau driven by mantle delamination from the overriding plate

In the format provided by the authors and unedited

This PDF file includes:

Supplementary Figures 1-6

Supplementary Tables 1-3

Supplementary References

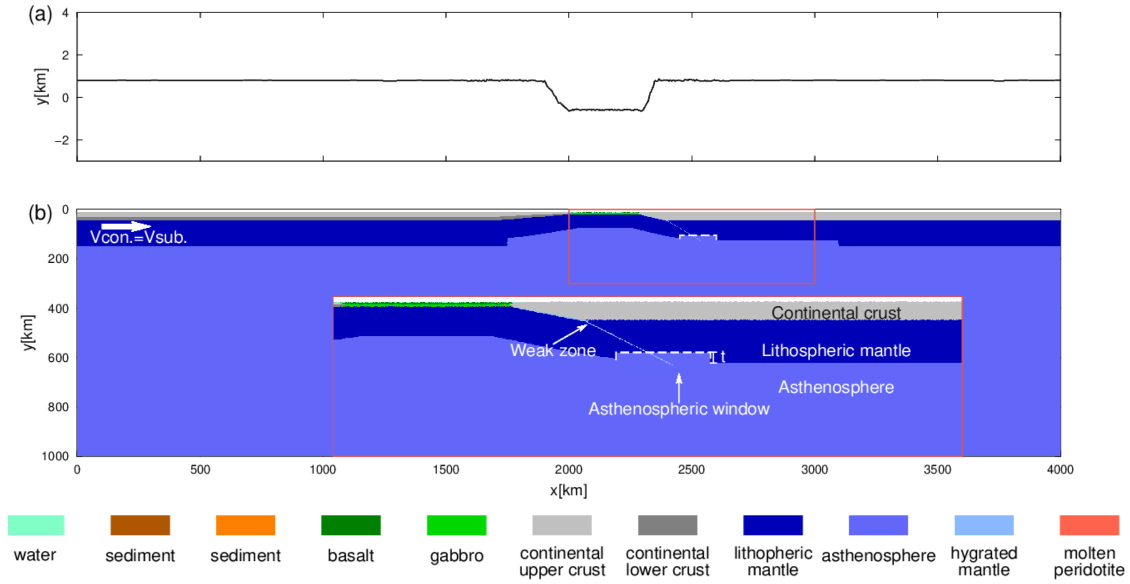

Figure S 1: (a) The initial topography. (b) The initial rock composition. The white dashed line shows the range of the asthenospheric window, and 't' indicates the thickness of the asthenospheric window. A detailed description is provided in the Methods section.

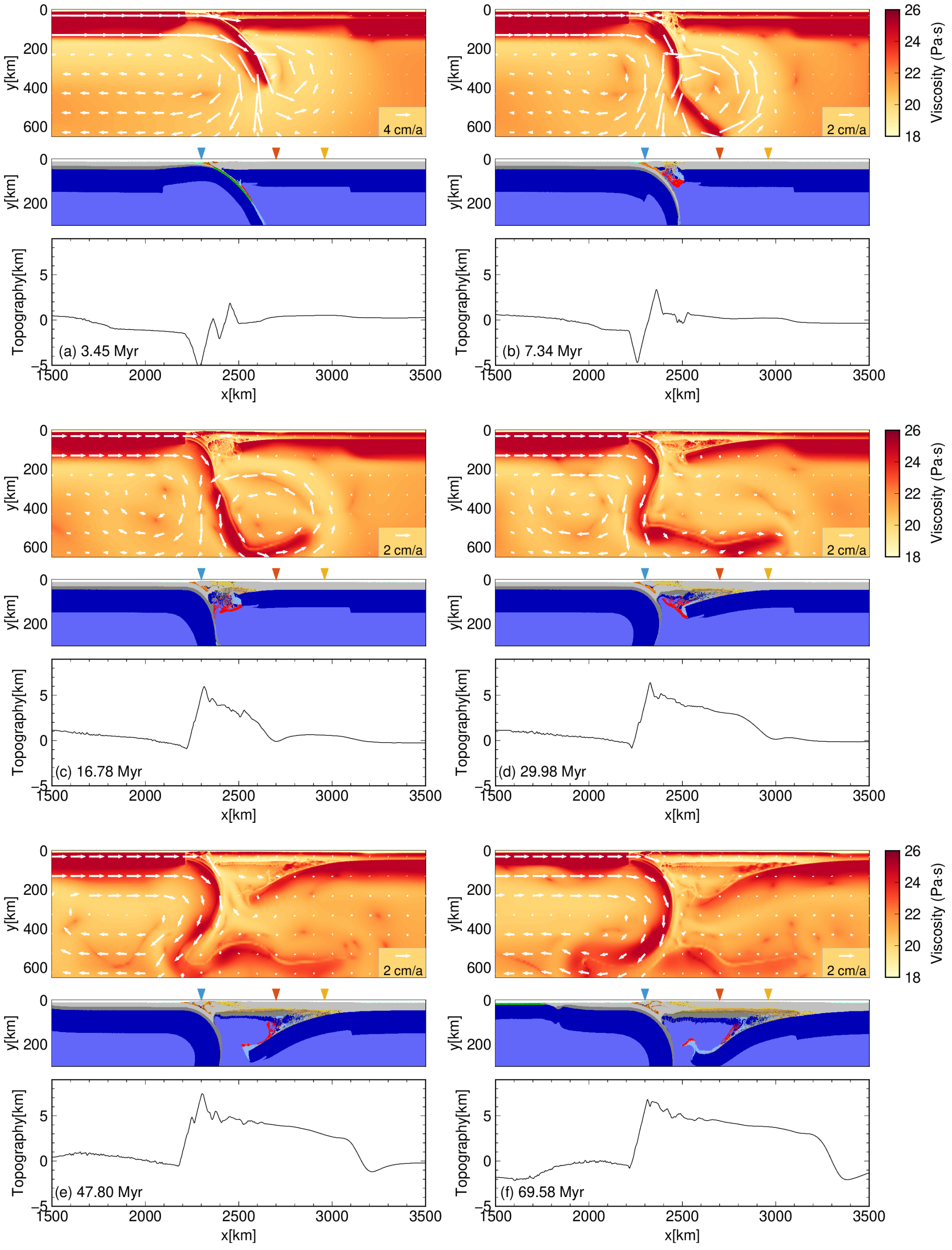

Figure S 2: Evolution of overriding plate mantle delamination shown by the modelled effective viscosity section overlain by the velocity vector, rock composition and topography. The uplift histories of three distinct positions, indicated by blue, orange and yellow triangles are shown in Fig. 4 and Fig. S6.

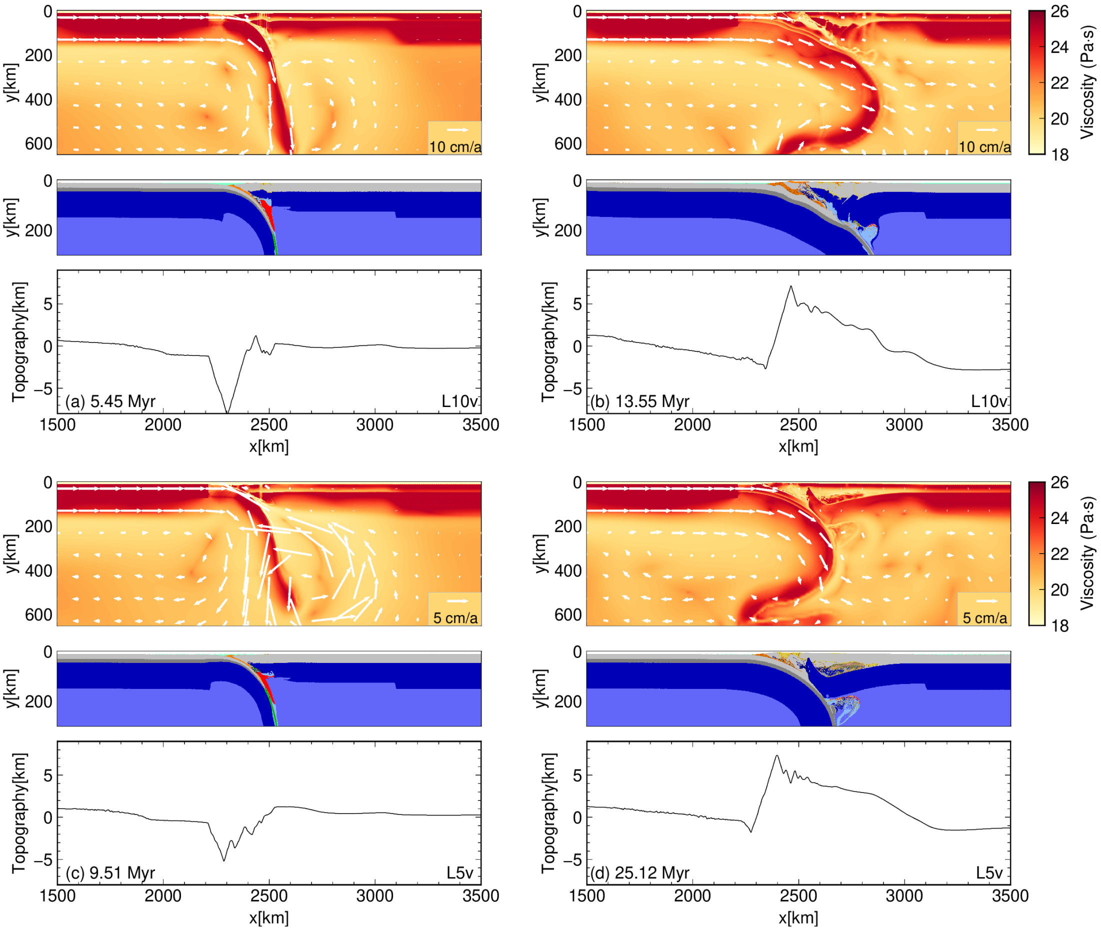

Figure S 3: Model predictions with constant convergence velocity. (a) and (b): Model with a constant convergence velocity of 10 cm/a on the subducting plate; (c) and (d): Model with a constant convergence velocity of 5 cm/a on the subducting plate.

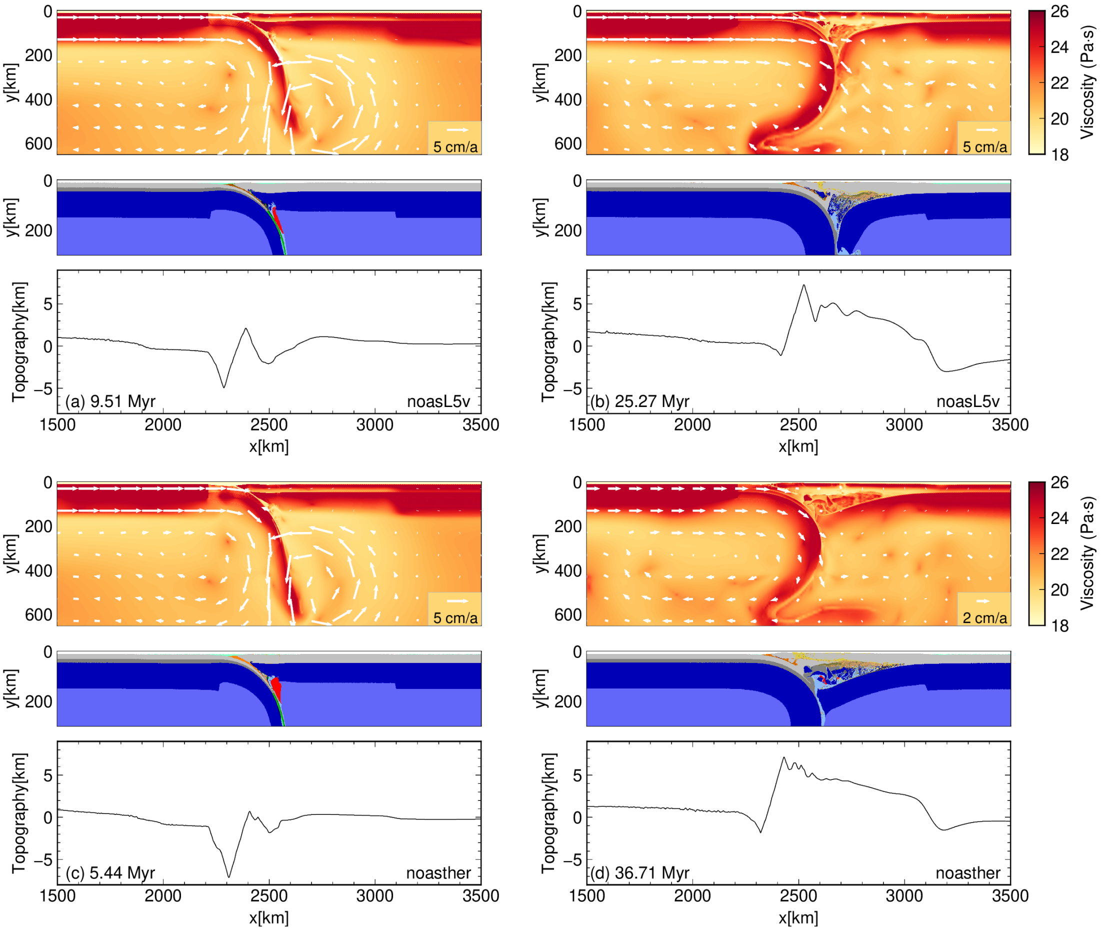

Figure S 4: Model predictions without a prescribed asthenospheric window. (a) and (b): Model without a prescribed asthenospheric window, while with a constant convergence velocity of 5 cm/a on the subducting plate; (c) and (d): Model without a prescribed asthenospheric window, while with the same convergence rate as the reference model of 10-4.5-2 cm/a.

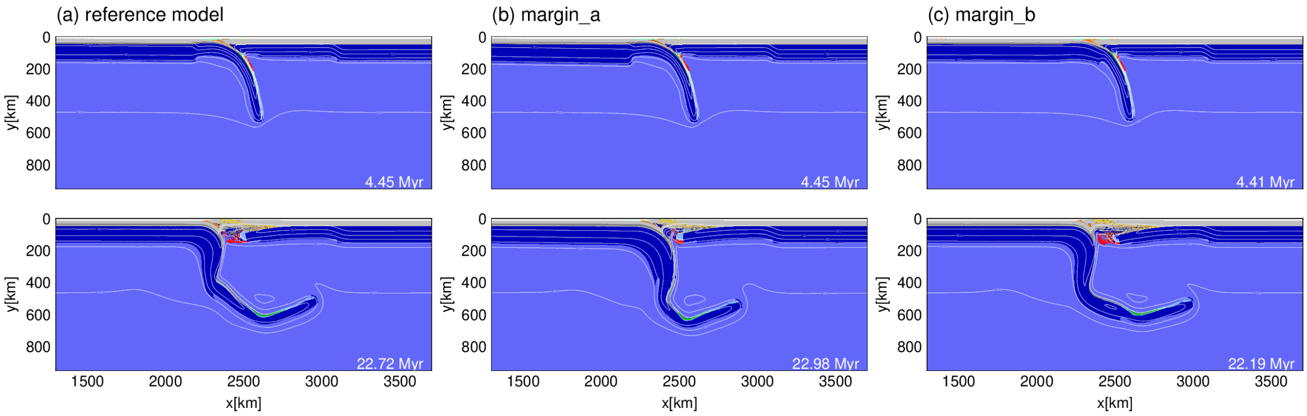

Figure S 5: Model results with different initial rifted margin thermal fields. All the models show the same process of overriding plate mantle delamination. A higher rifted margin geotherm leads to increased melting in the mantle wedge.

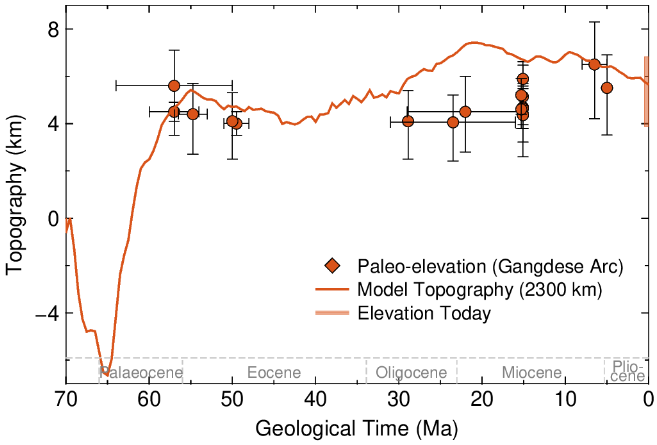

Figure S 6: Topography evolution of mantle delamination at 2300 km and comparison with paleo-elevations in the Gangdese arc. The red circles with error bars show the paleo-elevations in the Gangdese arc[1–6]. The solid line indicates the modelled topographic evolution at x = 2300 km, which corresponds to the Gangdese region.

Table S 1: Material rheology parameters

Material	Sediment	Felsic continental crust	Lower continental crust	Mantle (dry olivine)	Upper oceanic crust (altered basalt)	Lower oceanic crust (gabbro)
Density, ρ_0 (kg/m^3)	2600	2750	3000	3300	3000	3000
Pre-exponential factor, 1/AD (Pa^n s)	1.97×10^{17}	1.97×10^{17}	4.8×10^{22}	3.98×10^{16}	1.97×10^{17}	4.8×10^{22}
Activation energy, E (kJ/mol)	154	154	238	532	154	238
Power law exponent, n	2.3	2.3	3.2	3.5	2.3	3.2
Coefficients of friction, sin(φ)	0.3	0.3	0.3	0.3-0.1	0.3	0.6
Radioactive heat production, Hr (μW/m^3)	1.5	1	0.25	0.022	0.25	0.25

Table S 2: Main model parameters

Model Name	Input Parameters			Results
	Convergence Rate (cm/a)	$L_{\text{Oceanic Plate}}$ (km)	$t_{\text{Asthe. window}}$ (km)	
C2ALOL	10 - 4.5 - 2	275	70	Mature delamination
C3ALOL	10 - 4.5 - 3	275	70	Mature delamination
C4ALOL	10 - 4.5 - 4	275	70	Double-sided subduction
C2ALOM	10 - 4.5 - 2	475	70	Mature delamination
C3ALOM	10 - 4.5 - 3	475	70	Stalled delamination
C4ALOM	10 - 4.5 - 4	475	70	Stalled delamination
C2ALOH	10 - 4.5 - 2	675	70	Mature delamination
C3ALOH	10 - 4.5 - 3	675	70	Stalled delamination
C4ALOH	10 - 4.5 - 4	675	70	Stalled delamination
C2AMOL	10 - 4.5 - 2	275	45	Mature delamination
C3AMOL	10 - 4.5 - 3	275	45	Stalled delamination
C4AMOL	10 - 4.5 - 4	275	45	Double-sided subduction
C2AMOM	10 - 4.5 - 2	475	45	Mature delamination
C3AMOM	10 - 4.5 - 3	475	45	Stalled delamination
C4AMOM	10 - 4.5 - 4	475	45	Stalled delamination
C2AMOH	10 - 4.5 - 2	675	45	Stalled delamination
C3AMOH	10 - 4.5 - 3	675	45	Stalled delamination
C4AMOH	10 - 4.5 - 4	675	45	Double-sided subduction
C2AHOL (reference model)	10 - 4.5 - 2	275	20	Mature delamination
C3AHOL	10 - 4.5 - 3	275	20	Stalled delamination
C4AHOL	10 - 4.5 - 4	275	20	Stalled delamination
C2AHOM	10 - 4.5 - 2	475	20	Mature delamination
C3AHOM	10 - 4.5 - 3	475	20	Stalled delamination
C4AHOM	10 - 4.5 - 4	475	20	Double-sided subduction
C2AHOH	10 - 4.5 - 2	675	20	Double-sided subduction
C3AHOH	10 - 4.5 - 3	675	20	Stalled delamination
C4AHOH	10 - 4.5 - 4	675	20	Double-sided subduction
L5V	5	275	20	Stalled delamination
L10V	10	275	20	Accretion model
noasther	10 - 4.5 - 2	275	0	Stalled delamination
noasL5v	5	275	0	Double-sided subduction

$L_{\text{Oceanic Plate}}$ is the length of the oceanic plate; $t_{\text{Asthe. window}}$ is the thickness of the asthenospheric window.

Table S 3: Paleo-altitude data compilation

Time (Ma)	Paleo-altitude (m)	Reference
	**Northern Lhasa**	
33.9-56	4050(+1420/-1220)	[7]
23.03-37.71	4850(+1630/-1435)	[7]
33.9-56	3800(±1500)	[8]
5.33-23.03	4260(+475/-575)	[7]
5.33-23.03	4700(±1300)	[8]
19.8-25.5	3190(±100)	[9]
19.8-25.5	2770(±530)	[10]
19.8-25.5	3040(±560)	[10]
25.5	<2300	[11]
29-37	4100(±400)	[12]
26	4750(±250)	[13]
36-39	Sea level	[14]
46	2950(+910/-730)	[15]
26.5-21.5	3823(+1025/-1699)	[16]
26.5-21.5	4372(+1234/-1895)	[16]
	**Hoh-Xil Basin**	
35-40	2040(+1460/-1130)	[17]
35-55	1300(±400)	[8]
35-55	4850(±550)	[8]
37-40	<2000	[18]
5.33-23.03	3500(±100)	[8]
5.33-23.03	7850(±150)	[8]
30.8-37.8	2700(±200)	[19]
23.03-37.71	4000	[20]
17	2163(±768)	[21]
	**Gangdese arc**	
5	5509(+1390/-1993)	[1]
15	5200(+1370/-605)	[2]
15	5136(+1339/-1913)	[1]
15	4689(±895)	[3]
15	4638(±847)	[3]
15	5400(±728)	[4]
25-20	4500(+1500/-1700)	[5]
29-25	4100(+1300/-1600)	[5]
31-16	4057(+1154/-1640)	[1]
46-40	5600(+1500/-2100)	[5]
60-54	4500(±400)	[6]
51-48	4000(±500)	[6]
56-54	4400(+1300/-1700)	[5]
54-50	4100(+1200/-1600)	[5]

References

[1] Currie, B. S. *et al.* Multiproxy paleoaltimetry of the Late Oligocene-Pliocene Oiyug Basin, southern Tibet. *American Journal of Science* **316**, 401–436 (2016).

[2] Currie, B. S., Rowley, D. B. & Tabor, N. J. Middle Miocene paleoaltimetry of southern Tibet: Implications for the role of mantle thickening and delamination in the Himalayan orogen. *Geology* **33**, 181 (2005).

[3] Spicer, R. A. *et al.* Constant elevation of southern Tibet over the past 15 million years. *Nature* **421**, 622–624 (2003).

[4] Khan, M. A. *et al.* Miocene to Pleistocene floras and climate of the eastern Himalayan siwaliks, and new palaeoelevation estimates for the Namling–Oiyug Basin, Tibet. *Global and Planetary Change* **113**, 1–10 (2014).

[5] Ingalls, M. *et al.* Paleocene to Pliocene low-latitude, high-elevation basins of southern Tibet: Implications for tectonic models of India-Asia collision, Cenozoic climate, and geochemical weathering. *GSA Bulletin* **130**, 307–330 (2018).

[6] Ding, L. *et al.* The Andean-type Gangdese mountains: Paleoelevation record from the Paleocene–Eocene Linzhou Basin. *Earth and Planetary Science Letters* **392**, 250–264 (2014).

[7] Rowley, D. B. & Currie, B. S. Palaeo-altimetry of the Late Eocene to Miocene Lunpola Basin, central Tibet. *Nature* **439**, 677–681 (2006).

[8] Polissar, P. J., Freeman, K. H., Rowley, D. B., McInerney, F. A. & Currie, B. S. Paleoaltimetry of the Tibetan Plateau from D/H ratios of lipid biomarkers. *Earth and Planetary Science Letters* **287**, 64–76 (2009).

[9] Sun, J. M. *et al.* Palynological evidence for the latest Oligocene-early Miocene paleoelevation estimate in the Lunpola Basin, central Tibet. *Palaeogeography, Palaeoclimatology, Palaeoecology* **399**, 21–30 (2014).

[10] Jia, G. D., Bai, Y., Ma, Y. J., Sun, J. M. & Peng, P. A. Paleoelevation of Tibetan Lunpola Basin in the Oligocene–Miocene transition estimated from leaf wax lipid dual isotopes. *Global and Planetary Change* **126**, 14–22 (2015).

[11] Su, T. *et al.* No high Tibetan Plateau until the Neogene. *Science Advances* **5**, eaav2189 (2019).

[12] Xiong, Z. Y. *et al.* The rise and demise of the Paleogene Central Tibetan Valley. *Science Advances* **8**, eabj0944 (2022).

[13] DeCelles, P. G. *et al.* High and dry in central Tibet during the Late Oligocene. *Earth and Planetary Science Letters* **253**, 389–401 (2007).

[14] Wei, Y. *et al.* Low palaeoelevation of the northern Lhasa Terrane during Late Eocene: Fossil foraminifera and stable isotope evidence from the Gerze Basin. *Scientific Reports* **6**, 27508 (2016).

[15] Xu, Q., Ding, L., Hetzel, R., Yue, Y. H. & Rades, E. F. Low elevation of the northern Lhasa terrane in the Eocene: Implications for relief development in south Tibet. *Terra Nova* **27**, 458–466 (2015).

[16] Ingalls, M., Rowley, D. B., Currie, B. S. & Colman, A. S. Reconsidering the uplift history and peneplanation of the northern Lhasa terrane, Tibet. *American Journal of Science* **320**, 479–532 (2020).

[17] Cyr, A. J., Currie, B. S. & Rowley, D. B. Geochemical evaluation of Fenghuoshan group lacustrine carbonates, north-central Tibet: Implications for the paleoaltimetry of the Eocene Tibetan Plateau. *The Journal of Geology* **113**, 517–533 (2005).

[18] Miao, Y. F. *et al.* A Late-Eocene palynological record from the Hoh Xil Basin, northern Tibetan Plateau, and its implications for stratigraphic age, paleoclimate and paleoelevation. *Gondwana Research* **31**, 241–252 (2016).

[19] Song, B. W. *et al.* Reconstruction of the latest Eocene-early Oligocene paleoenvironment in the Hoh Xil Basin (central Tibet) based on palynological and ostracod records. *Journal of Asian Earth Sciences* **217**, 104860 (2021).

[20] Lin, J. *et al.* Late Eocene–Oligocene high relief paleotopography in the north central Tibetan Plateau: Insights from detrital zircon U–Pb geochronology and leaf wax hydrogen isotope studies. *Tectonics* **39**, e2019TC005815 (2020).

[21] Sun, B. *et al.* Early Miocene elevation in northern Tibet estimated by palaeobotanical evidence. *Scientific Reports* **5**, 10379 (2015).
